# A Comparative Analysis of Ursolic and Oleanolic Acids in Eleven *Epilobium* Species

**DOI:** 10.3390/molecules31091510

**Published:** 2026-05-01

**Authors:** Kateryna Uminska, Zigmantas Gudžinskas, Victoriya Georgiyants, Liudas Ivanauskas, Mindaugas Marksa, Viktoriia Volochai, Alla Kozurak, Olha Mykhailenko

**Affiliations:** 1 Zhytomyr Basic Pharmaceutical Professional College, Chudnivska Str. 99, 10005 Zhytomyr, Ukraine; uminska.kateryna@pharm.zt.ua; 2 State Scientific Research Institute Nature Research Centre, Laboratory of Flora and Geobotany, LT-08412 Vilnius, Lithuania; 3 Pharmaceutical Chemistry Department, National University of Pharmacy, 61168 Kharkiv, Ukraine; vgeor@nuph.edu.ua (V.G.);; 4 Department of Analytical and Toxicological Chemistry, Lithuanian University of Health Sciences, LT-50161 Kaunas, Lithuania; liudas.ivanauskas@lsmu.lt (L.I.); mindaugas.marksa@lsmu.lt (M.M.); 5 Department of Pharmacognosy, National University of Pharmacy, 61002 Kharkiv, Ukraine; v.volochai@pdmu.edu.ua; 6 Carpathian Biosphere Reserve, 90600 Rakhiv, Ukraine; cbr-rakhiv@ukr.net; 7 Pharmacognosy and Phytotherapy Group, UCL School of Pharmacy, London WC1N 1AX, UK; 8 Department of Pharmaceutical Biology, Kiel University, 24118 Kiel, Germany

**Keywords:** pentacyclic triterpenoid, oleanolic acid, ursolic acid, genus *Epilobium* L.

## Abstract

A comparative analysis of pentacyclic triterpenoids (ursolic acid and oleanolic acid) in the aerial parts of eleven *Epilobium* species was performed using high-performance liquid chromatography (HPLC) results. Samples for the analysis were collected from various regions in Ukraine, Poland and Lithuania. Oleanolic acid and ursolic acid were identified and quantified in nine of the species (*E. angustifolium*, *E. montanum*, *E. collinum*, *E. roseum*, *E. palustre*, *E. tetragonum*, *E. obscurum*, *E. nervosum*, and *E. nutans*). However, neither compound was detected in *E. parviflorum* or *E. hirsutum* from any collection site, indicating notable chemotaxonomic divergence within the genus. The quantity of ursolic acid in the analysed samples ranged from 20.27 ± 0.49 to 74.84 ± 2.24 mg/100 g dry weight, consistently exceeding that of oleanolic acid (2.03 ± 0.05 to 32.09 ± 0.73 mg/100 g). The highest total triterpenoid content was observed in *E. tetragonum*. These findings emphasise the importance of oleanolic and ursolic acids as auxiliary chemotaxonomic markers for *Epilobium* species. Given the well-documented antiproliferative and antiviral activities of these triterpenoids, the present results also suggest that several under-explored *Epilobium* species could be a promising source of bioactive compounds for further pharmacological research, particularly regarding prostate cancer and viral infections.

## 1. Introduction

With approximately 165 species distributed across all continents except Antarctica, *Epilobium* L is the largest genus in the Onagraceae family [1]. In Europe, 77 species of this genus are considered native or naturalised, occupying a wide range of ecological habitats [2,3]. For centuries, various *Epilobium* species have been used as traditional remedies for inflammatory conditions, skin injuries and urinary tract disorders in Central and Eastern Europe, as well as parts of North America.

The aerial parts of several species are used to produce the traditional herbal remedy *Epilobii herba*, as recognised in regional pharmacopoeias [4]. A monograph on *Epilobii herba* is available in the Czech Pharmaceutical Codex of 1993. This includes the dried flowering herb of *Epilobium parviflorum*, *E. montanum*, *E. collinum* and *E. roseum* [5]. In Hungary, tablets containing *E. parviflorum* herb for oral use were registered as herbal medicinal products from 1999 to 2013 [6,7]. However, the traditional medicine *Epilobii herba* may also contain large-flowered species, such as *E. angustifolium* L. (=*Chamerion angustifolium* (L.) Holub) and *E. hirsutum* L. [4].

*Epilobium angustifolium* is a widespread and highly variable species within the genus [5,8]. It has a circumboreal distribution, extending from the Arctic or subarctic regions southwards into cool temperate regions in North America and Eurasia [1]. It is widespread throughout Ukraine, growing in deciduous and mixed forests, clearings and areas affected by forest fires [9].

A growing number of pharmacological studies have confirmed the traditional use of *Epilobium* species for treating urinary disorders and prostate conditions [6,10,11,12]. Numerous in vitro and in vivo studies have demonstrated the antiproliferative, anti-inflammatory, antioxidant, antimicrobial and antiviral properties of *Epilobium* extracts [13,14,15,16]. This biological activity is primarily due to the presence of macrocyclic ellagitannins, such as oenothein B, as well as flavonoids, phenolic acids, steroids, triterpenoids and amino acids [17,18,19,20].

Pentacyclic triterpenoids, particularly ursolic and oleanolic acids, are attracting increasing attention due to their well-documented antiproliferative, anti-inflammatory and antiviral properties [21,22,23]. Both compounds have been reported to inhibit the survival and proliferation of human prostate cancer cells, supporting their relevance in prostate disease research [24,25]. However, knowledge of their accumulation and quantity in *Epilobium* species remains limited. Previous studies have predominantly identified ursolic and oleanolic acids in *E. angustifolium* [26], while comprehensive data on other species is lacking.

Furthermore, comparative chemotaxonomic studies assessing the distribution of these triterpenoids among various *Epilobium* species, particularly those found in Eastern Europe, are lacking. Given the high diversity of the genus in Ukraine and neighbouring regions [27,28,29], and the absence of systematic data on the triterpene profiles of these species, further research seems necessary. Improving our understanding of the distribution and quantification of triterpenoids in these plants could facilitate not only chemotaxonomic characterisation, but also the identification of new sources of bioactive compounds with potential pharmacological significance.

The aim of this study was to conduct a comparative analysis of ursolic and oleanolic acids in the aerial parts of eleven *Epilobium* species collected in Ukraine, Poland and Lithuania. The study sought to expand our current understanding of the distribution of these acids within the genus, and to explore the potential of understudied *Epilobium* species as sources of bioactive pentacyclic triterpenoids. In this study, we sought to answer the following questions: (a) What are the qualitative and quantitative differences in pentacyclic triterpenoids among the studied *Epilobium* species? (b) How does the content of pentacyclic triterpenoids differ among *E. angustifolium* populations from different regions of Eastern Europe? (c) What environmental factors influence the content of pentacyclic triterpenoids in the raw material of *Epilobium* species? (d) What is the pharmacological potential of Epilobium species as a source of triterpenoids?

## 2. Results

### Pentacyclic Triterpenoids in Epilobium Species

**Figure 1:**
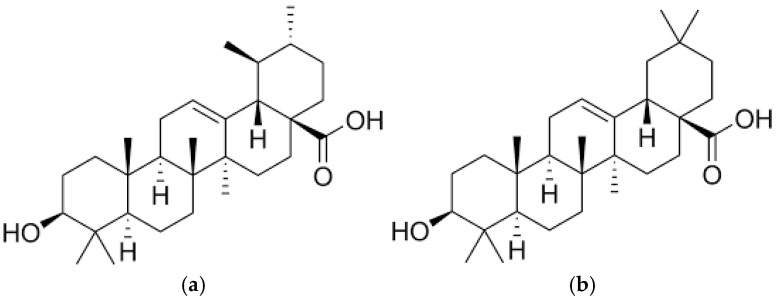
Structural formulas of ursolic (**a**) and oleanolic (**b**) acids, identified in *Epilobium* species.

Results from HPLC analysis of the aerial parts of eleven *Epilobium* species collected in Ukraine, Lithuania and Poland revealed substantial variability in the content of pentacyclic triterpenoids (oleanolic acid and ursolic acid) among the species studied (Figure 1). Eight of the ten species studied (*E. angustifolium*, *E. collinum*, *E. montanum*, *E. nervosum*, *E. nutans*, *E. obscurum*, *E. palustre* and *E. tetragonum*) were found to contain both compounds; however, for most species (n = 1), these observations are based on single-sample analysis and should be considered preliminary. In contrast, *E. hirsutum* and *E. parviflorum* exhibited a total absence of both oleanolic acid and ursolic acid at all collection sites. Furthermore, oleanolic acid was not detected in the analysed *E. roseum* sample (Table 1).

**Figure 2:**
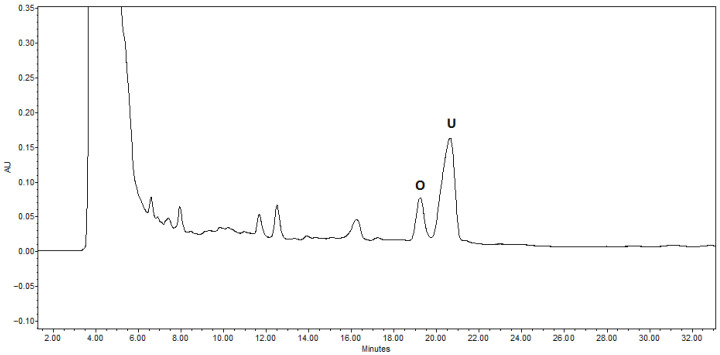
Representative HPLC-DAD chromatograms (λ = 205 nm) of triterpenoid acids of *E. angustifolium* (E_ang2). Peak assignments: O—oleanolic acid; U—ursolic acid.

The current study of eleven *Epilobium* species reveals a wide range of variability. Ursolic acid content ranged from 20.27 ± 0.49 mg/100 g dw to 216.31 ± 101.61 mg/100 g dw (equivalent to 0.20–0.75 mg/g dw), with the highest content observed in *E. angustifolium*. Oleanolic acid was present in smaller quantity, ranging from 2.03 ± 0.05 mg/100 g dw to 32.09 ± 0.73 mg/100 g dw (i.e., 0.02–0.32 mg/g dw). The HPLC metabolite profiling analysis of *E. angustifolium* samples revealed the presence of triterpenoids, as shown in Figure 2.

**Figure 3:**
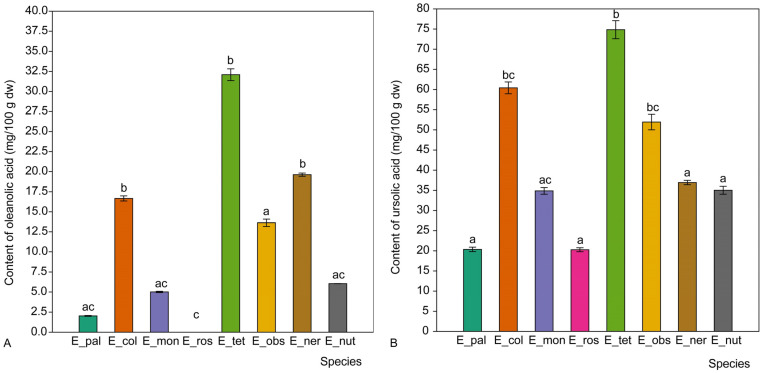
The mean content (in mg/100 g of dry weight) of oleanolic acid (**A**) and ursolic acid (**B**) in the raw plant material of *Epilobium* sect. *Epilobium* species. Abbreviations of species: E_pal—*E. palustre*, E_col—*E. collinum*, E_mon—*E. montanum*, E_ros—*E. roseum*, E_tet—*E. tetragonum*, E_obs—*E. obscurum*, E_ner—*E. nervosum*, E_nut—*E. nutans*. Different lowercase letters above the bars indicate significant differences according to the Dunn‘s post hoc test (*p* < 0.05).

The highest concentration of oleanolic acid (Figure 3) was found in *E. tetragonum*, based on a single analysed sample, which was significantly higher than in the other species studied, except for *E. nervosum* and *E. collinum*. The same trend was observed for ursolic acid, with *E. tetragonum* having a significantly higher content (*p* < 0.01) than in most of the other species studied, except for *E. nervosum* and *E. collinum*.

Although *E. angustifolium* was previously shown to have a high triterpenoid content [19], some species, such as *E. tetragonum* and *E. collinum*, also displayed higher acid content. This indicates that less-studied species may serve as an alternative source of bioactive pentacyclic triterpenoids.

Significant differences in oleanolic acid (H = 28.74; *p* < 0.001) and ursolic acid (H = 28.29; *p* < 0.001) content were found among the ten *E. angustifolium* samples studied (Table 2). The mean oleanolic acid content of *E. angustifolium* samples was 35.09 ± 20.56 mg/100 g dw. The sample from Lithuania (E_ang9; open hill slope habitat) had the highest oleanolic acid content (72.00 ± 1.58 mg/100 g dw), while the sample from the nearby locality (semi-shaded forest edge habitat) had the lowest content (6.81 ± 0.11 mg/100 g dw). The mean content of ursolic acid in samples of *E. angustifolium* was 216.31 ± 101.61 mg/100 g dw. The highest content (386.80 ± 4.77 mg/100 g dw) was found in a sample from an open hill slope in Lithuania (E_ang9), while the lowest content (63.35 ± 1.50 mg/100 g dw) was found in a sample from a semi-shaded forest edge in a nearby locality.

**Figure 4:**
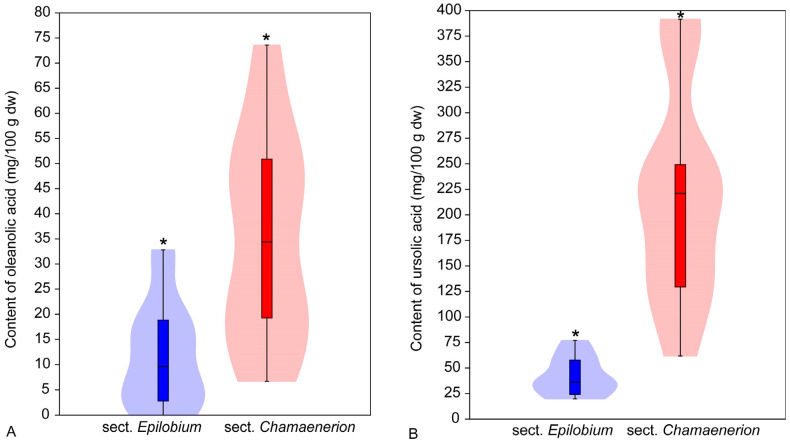
The mean content (in mg/100 g of dry weight) of oleanolic acid (**A**) and ursolic acid (**B**) in the raw plant material of *Epilobium* sect. *Epilobium* and *Epilobium* sect. *Chamaenerion*. The asterisk (*) above the violin indicates a significant difference between the datasets (*p* < 0.001).

The analysis revealed that plants of *Epilobium* sect. *Chamaenerion* (*E. angustifolium*) accumulate a significantly higher concentration of both oleanolic acid (H = 19.70; *p* < 0.001) and ursolic acid (H = 37.12; *p* < 0.001) than plants of *Epilobium* sect. *Epilobium* (all other species studied) (Figure 4).

Negative but insignificant correlations were found between the content of oleanolic and ursolic acids in *E. angustifolium* raw material and the altitude of the collection site (r_s_ = −0.18, *p* = 0.337 and r_s_ = −0.26, *p* = 0.160, respectively). This suggests that plants growing above sea level accumulate slightly less of the studied compounds. However, it is unclear what factors contribute most to their accumulation, and further studies are required.

A negative and significant correlation was found between oleanolic acid content (r_s_ = −0.44, *p* = 0.032) and a negative, though insignificant, correlation was found between ursolic acid content (r_s_ = −0.25, *p* = 0.244) in *Epilobium* species raw material and the altitude of the collection site. However, there is insufficient data to conclude whether elevation affects the accumulation of the analysed compounds or if this depends on the *Epilobium* species or other environmental factors.

## 3. Discussion

This study is the first assessment of pentacyclic triterpenoids, specifically ursolic acid and oleanolic acid, in different *Epilobium* species growing in Eastern Europe. Analysing eleven species collected under diverse geographical and environmental conditions expands our knowledge of the distribution of these bioactive compounds within the genus, revealing significant differences in triterpenoid profiles between species.

The results show that the presence and relative abundance of ursolic and oleanolic acids are significantly species-specific. Consistent accumulation was apparently observed in several taxa (e.g., *E. tetragonum*, *E. collinum* and *E. montanum*), although this observation is based on single-sample data for most of these species and requires further confirmation. Consistent accumulation was observed in all analysed samples of *E. parviflorum* and *E. hirsutum*, regardless of their geographic location. These findings tentatively suggest that the distribution of triterpenoids in *Epilobium* is primarily influenced by genetic and evolutionary factors rather than environmental conditions alone. This supports the potential use of these compounds as auxiliary chemotaxonomic markers within the genus [30,31]. Thus, the results of this study suggest that *E. parviflorum* and *E. hirsutum* belong to a distinct group, which is partly consistent with the results of morphological, anatomical, and genetic studies [32].

In our previous study of *E. angustifolium* collected in Ukraine [19], the content of triterpenoid acids varied depending on the plant organ and phenological stage. The highest concentration of ursolic acid was observed in the leaves, reaching 3.51 mg/g of dry weight during the early flowering stage, while the highest concentration of oleanolic acid was observed in the flowers, reaching 0.61 mg/g of dry weight during the early flowering stage. Overall, ursolic acid dominated oleanolic acid in all plant parts and stages.

Although these data indicate significant variation in triterpenoid accumulation within and between species, the present study examines the distribution patterns of ursolic and oleanolic acids to assess whether they reflect taxonomic relationships within the genus.

The fact that *E. angustifolium* growing in two nearby localities in the Plungė district of Lithuania accumulates very different amounts of oleanolic acid and ursolic acid suggests that the characteristics of the habitat are one of the most important factors influencing the accumulation of these compounds in plants. In this case, the amount of light received by the plants may be an important factor [33]. The highest concentration of the studied compounds was found in plants growing on a hillside in a warm, open habitat, while the lowest concentration was found in plants growing in semi-shade at the edge of a forest. This observation is consistent with previous reports [34,35] indicating that triterpenoid biosynthesis can be stimulated under conditions of increased solar radiation and abiotic stress, where these compounds may contribute to membrane stabilisation and stress adaptation. However, this interpretation should be treated with caution, given that other environmental factors, such as altitude, climate and soil conditions, also vary between sampling sites and were not systematically assessed in this study.

The consistent presence of ursolic acid and oleanolic acid in species such as *E. tetragonum*, *E. montanum,* and *E. collinum* may explain their traditional use in treating prostate-related diseases and inflammation [36,37]. However, such effects are probably due to the combined action of multiple compounds belonging to different phytochemical classes.

Pentacyclic triterpenoids, such as oleanolic and ursolic acids, exhibit a wide range of biological activities, including anti-cancer and antiviral effects [38]. Studies in vitro and in vivo show that both compounds inhibit the proliferation of prostate cancer cells and induce apoptosis via modulation of signalling pathways such as PI3K/Akt/mTOR [22,23], potentially enhancing the antiproliferative effects of ellagitannins present in *Epilobium* extracts.

Recent studies indicate that ursolic acid and oleanolic acid can inhibit the replication of viruses such as influenza, HSV, hepatitis B, and SARS-CoV-2 [21,25,39]. Therefore, *Epilobium* species containing these triterpenoids could be promising multi-target agents.

Quantifying ursolic acid and oleanolic acid in Ukrainian *Epilobium* species not only provides chemotaxonomic insight and supports their pharmacological relevance [36,37,38]. *Epilobium* species such as *E. tetragonum*, *E. collinum*, and *E. montanum* could be promising candidates for further pharmacological research. However, the concentrations of triterpenoids detected may be insufficient to produce biological effects in isolation and require further investigation. Conversely, the absence of these compounds in *E. parviflorum* and *E. hirsutum* indicates the necessity of investigating alternative bioactive metabolites in these species.

The established presence of oleanolic and ursolic acids in several *Epilobium* species highlights their potential pharmacological significance. These triterpenoids are known for their anti-inflammatory, antioxidant, and antiproliferative activities, suggesting that certain *Epilobium* taxa may serve as promising natural sources of bioactive compounds. However, further pharmacological and bioavailability studies are needed to fully evaluate their therapeutic potential.

Certainly, oleanolic and ursolic acids are present in *Epilobium* species in mixtures with other substances, so the activity of plant extracts is related to the synergistic effect of all components. This is a limitation of this study, and further research on both isolated compounds and whole extracts is needed to clarify the pharmacological effects.

There are several limitations to this study that should be noted. Analysis of most *Epilobium* species was performed on a limited number of samples, which restricts the assessment of intraspecific variability. Furthermore, only two pentacyclic triterpenoids were quantified, even though other structurally related compounds may also contribute to chemotaxonomic differentiation and biological activity. Nevertheless, the clear species-specific patterns of presence or absence observed across multiple sampling sites support the robustness of the main findings.

Overall, this investigation revealed that ursolic and oleanolic acids are widely distributed across different *Epilobium* species. This finding highlights the phytochemical diversity within the genus and supports the idea of investigating less-studied *Epilobium* taxa as potential sources of pharmacologically important triterpenoids.

## 4. Materials and Methods

### 4.1. Plant Material

The raw plant material of eleven *Epilobium* species belonging to *Epilobium* sect. *Chamaenerion* (*E. angustifolium* (L.) Scop.), as well as *Epilobium* sect. *Epilobium* (*E. parviflorum* Schreb., *E. hirsutum* L., *E. montanum* L., *E. collinum* C. C. Gmel., *E. roseum* Schreb, *E. palustre* L., *E. tetragonum* L., *E. obscurum* Schreb, *E. nervosum* Boiss. & Buhse and *E. nutans* F. W. Schmidt.), was analysed. The raw plant material was collected in Ukraine, Lithuania and Poland in June–July 2019 (Table 3). Plant material was collected in accordance with local regulations and, where required, appropriate permits for sampling in protected areas were obtained. All voucher specimens (OM2019-01–OM2019-21) verified by Dr. Mykhailenko and deposited at the Herbarium of the National University of Pharmacy in Ukraine. The raw material was dried at an ambient temperature of 20–24 °C and used for the chemical analysis. Dried samples of the raw material (10 g of each species) were crushed to obtain particles measuring 2–3 mm.

The nomenclature of the *Epilobium* species is based on Plants of the World Online [40] with one exception. While *Epilobium nervosum* Boiss. & Buhse is now commonly recognised as *Epilobium roseum* subsp. *subsessile* (Boiss.) P.H. Raven, but in this study, we treat it as a distinct species.

### 4.2. Preparation of Extracts

1.0 g of each powdered sample was filled with 1 mL 100% (*v*/*v*) acetone and subjected to ultrasound-assisted extraction for 30 min at room temperature. The extractive solutions were then centrifuged for 30 min at 3000× *g* in a Biofuge Stratos centrifuge. The obtained extracts were filtered through 0.22 µm syringe filters (Carl RothGmbH & Co. KG, Karlsruhe, Germany) and stored at 4 °C until the analysis.

### 4.3. HPLC Analysis

Ursolic acid and oleanolic acid were separated using a Waters e2695 Alliance HPLC system coupled with a 2998 PDA detector (Waters, Milford, MA, USA) on an ACE Super C_18_ (250 mm × 4.6 mm, 3 µm) column (ACT, Aberdeen, UK). The mobile phase consisted of methanol and water (90/10, *v*/*v*). The flow rate was 0.6 mL/min, with an injection volume of 10 µL. Absorption was measured at 203 nm. Quantification was performed using the external standard method and calibration curves were obtained (oleanolic acid R^2^ = 0.999383; ursolic acid R^2^ = 0.998872) [41].

### 4.4. Statistical Analysis

All analyses of each sample were repeated three times, and the data from the HPLC analysis were processed using the LabSolutions Analysis Data System (Shimadzu). As neither oleanolic acid nor ursolic acid was detected in the *E. hirsutum* and *E. parviflorum* raw material, these species were excluded from further analysis. Descriptive statistics included the mean and standard deviation. Since most data sets were small or not normally distributed, non-parametric tests were applied. Comparisons between datasets were performed using the Kruskal–Wallis test, whereas comparisons between pairs of datasets were performed using the Dunn’s post hoc test. Statistical significance was defined as *p* < 0.05. The Spearman rank correlation method was used to calculate the correlation between the analysed factors. The statistical analyses were performed, and the graphs were drawn using PAST 5.1 software [42].

## 5. Conclusions

Ursolic acid and oleanolic acid were first identified in the analysed samples of *E. angustifolium*, *E. palustre*, *E. collinum*, *E. montanum*, *E. tetragonum*, *E. obscurum*, *E. nervosum*, and *E. nutans*. The aerial parts of *E. roseum* differed from those of the other species by containing the lowest level of ursolic acid (20.27 ± 0.49 mg/100 g dw) and by lacking oleanolic acid. By contrast, *E. parviflorum* and *E. hirsutum* were completely devoid of these triterpenoids in all samples tested. These findings suggest that ursolic acid and oleanolic acid could be used as additional markers for classifying species within the *Epilobium* genus and could inform future studies on species differentiation and the distribution of bioactive compounds. However, it should be noted that the results for most species are based on single-sample analyses. Therefore, these findings should be considered preliminary and require confirmation through larger-scale sampling.

## Figures and Tables

**Table 1:** Content of ursolic acid and oleanolic acid (mg/100 g of dry weight; mean ± standard deviation) in the raw material of the studied *Epilobium* species (*n* is the number of analysed samples).

Species	*n*	Oleanolic Acid	Ursolic Acid
*Epilobium angustifolium* L.	10	35.09 ± 20.56	216.31 ± 101.61
*Epilobium collinum* C.C.Gmel.	1	16.67 ± 0.32	60.42 ± 1.47
*Epilobium hirsutum* L.	3	0	0
*Epilobium montanum* L.	1	5.01 ± 0.08	34.86 ± 0.83
*Epilobium nervosum* Boiss. & Buhse	1	19.62 ± 0.20	36.94 ± 0.54
*Epilobium nutans* F.W.Schmidt	1	6.05 ± 0.01	35.02 ± 0.99
*Epilobium obscurum* Schreb.	1	13.63 ± 0.46	51.94 ± 1.93
*Epilobium palustre* L.	1	2.02 ± 0.05	20.36 ± 0.55
*Epilobium parviflorum* Schreb.	3	0	0
*Epilobium roseum* (Schreb.) Schreb.	1	0	20.27 ± 0.49
*Epilobium tetragonum* L.	1	32.09 ± 0.73	74.84 ± 2.24

**Table 2:** The content (in mg/100 g of dw) of oleanolic acid and ursolic acid (mean ± standard deviation) in the samples of the studied *Epilobium angustifolium* raw material.

Samples	Country of Origin	Oleanolic Acid	Ursolic Acid
E_ang1	Ukraine	20.70 ± 0.59	127.80 ± 4.40
E_ang2	Ukraine	50.50 ± 1.47	235.90 ± 5.19
E_ang3	Ukraine	11.30 ± 0.37	218.90 ± 5.67
E_ang4	Ukraine	44.80 ± 0.94	223.10 ± 6.31
E_ang5	Ukraine	41.80 ± 1.17	247.50 ± 6.70
E_ang6	Ukraine	19.40 ± 0.64	125.90 ± 4.19
E_ang7	Poland	27.10 ± 1.04	156.00 ± 4.22
E_ang8	Lithuania	56.50 ± 0.61	377.90 ± 6.97
E_ang9	Lithuania	72.00 ± 1.58	386.80 ± 4.77
E_ang10	Lithuania	6.81 ± 0.11	63.35 ± 1.50

**Table 3:** Sampling sites for the raw material of the *Epilobium* species in 2019.

Species	Site Code	Geographical Coordinates	Location	Elevation (m a.s.l.)	Date of Sampling
*E. palustre*	E_pal	48.16284° N; 24.38789° E	Ukraine, Zakarpattia region, Carpathian Mountains, Chornahora massif	1670	24 July
*E. collinum*	E_col	48.16735° N; 24.32771° E	Ukraine, Zakarpattia region, Carpathian Mountains, Chornahora massif	1206	23 July
*E. montanum*	E_mon	48.15674° N; 24.35087° E	Ukraine, Zakarpattia region, Carpathian Mountains, Chornahora massif	1340	24 July
*E. roseum*	E_ros	49.79887° N; 23.87778° E	Ukraine, Lviv region, Obroshyne village	335	12 July
*E. tetragonum*	E_tet	48.16523° N; 24.28228° E	Ukraine, Zakarpattia region, Carpathian Mountains, Kvasy village	538	25 July
*E. obscurum*	E_obs	49.79755° N; 23.87631° E	Ukraine, Lviv region, Obroshyne village	328	17 July
*E. nervosum*	E_ner	51.57568° N; 23.97800° E	Ukraine, Volyn region, Mel’nyky village	157	20 June
*E. nutans*	E_nut	48.16284° N; 24.38789° E	Ukraine, Zakarpattia region, Carpathian Mountains, Chornahora massif	1670	24 July
*E. hirsutum*	E_hir1	48.21078° N; 24.30931° E	Ukraine, Zakarpattia region, Carpathian Mountains, Kvasy village	641	23 July
*E. hirsutum*	E_hir2	50.01787° N; 36.30148° E	Ukraine, Kharkiv region, city Kharkiv	108	4 July
*E. hirsutum*	E_hir3	49.91099° N; 36.18951° E	Ukraine, Kharkiv region, Pokotilovka village	118	5 July
*E. parviflorum*	E_par1	49.62435° N 36.32810° E	Ukraine, Kharkiv region Gaidary village	93	3 July
*E. parviflorum*	E_par2	51.49161° N; 23.87142° E	Ukraine, Volyn region, Svityaz village	164	15 July
*E. parviflorum*	E_par3	48.15644° N; 24.34987° E	Ukraine, Zakarpattia region, Carpathian Mountains, Chornahora massif	1342	4 July
*E. angustifolium*	E_ang1	48.04715° N; 24.63105° E	Ukraine, Zakarpattia region, Carpathian Mountains, Chornahora massif	1958	23 June
*E. angustifolium*	E_ang2	48.15656° N; 24.33731° E	Ukraine, Zakarpattia region, Carpathian Mountains, Chornahora massif	1203	22 July
*E. angustifolium*	E_ang3	48.08450° N; 24.16812° E	Ukraine, Zakarpattia region, Carpathian Mountains, Kuziy-Trybushansky massif	977	18 July
*E. angustifolium*	E_ang4	49.66174° N; 24.27277° E	Ukraine, Lviv region, Shpilchina village	376	26 June
*E. angustifolium*	E_ang5	49.41504° N; 34.58589° E	Ukraine, Poltava region, Pisaivka village	101	16 June
*E. angustifolium*	E_ang6	50.00832° N; 35.21048° E	Ukraine, Kharkiv region, Kachalivka village	140	22 June
*E. angustifolium*	E_ang7	52.01133° N; 20.72603° E	Poland, Mazovian district, Żabia Wola area, Ojrzanów Towarzystwo village	149	9 July
*E. angustifolium*	E_ang8	54.64413° N; 24.06725° E	Lithuania, Prienai district, Pociūnai village	54	10 July
*E. angustifolium*	E_ang9	55.97992° N; 21.90638° E	Lithuania, Plungė district, Pauošniai village, Žemaitija National Park	160	14 July
*E. angustifolium*	E_ang10	55.97998° N; 21.90666° E	Lithuania, Plungė district, Pauošniai village, Žemaitija National Park	160	14 July

## Data Availability

All data supporting the results of this study are included in the manuscript, and the datasets are available upon request.
